# Supplementary material for: Forgetting Enhances Episodic Control With Structured Memories
Source: Front Comput Neurosci. 2022 Mar 25;16:757244. doi: 10.3389/fncom.2022.757244 (PMC8991683; doi:10.3389/fncom.2022.757244)
Supplement: Supplementary file 1 [file Data_Sheet_1.pdf]

Supplementary Material

1 SUPPLEMENTARY TABLES AND FIGURES

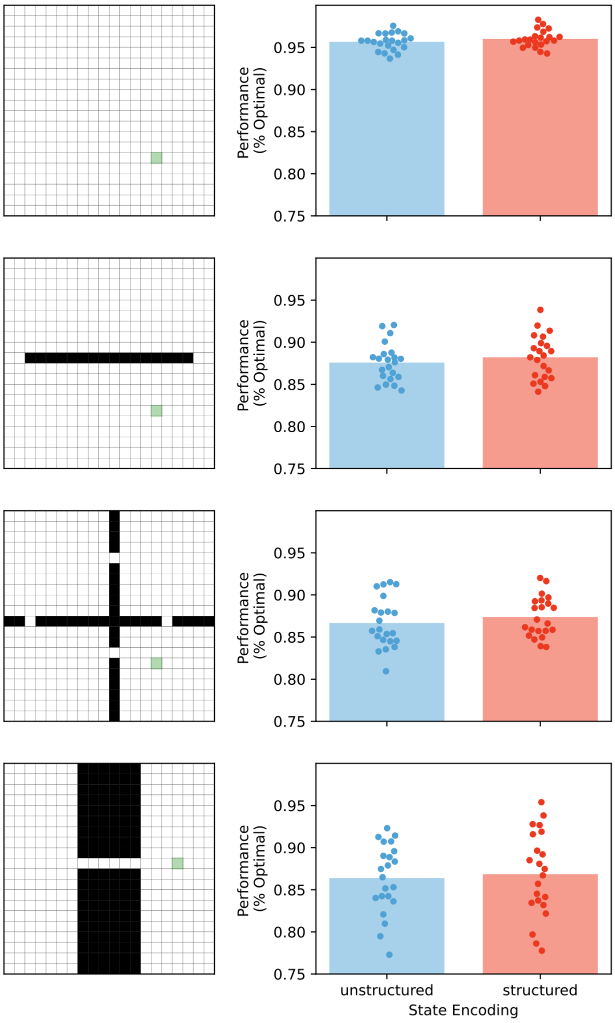

Figure S1. Average performance for episodic control using structured and unstructured representations with unrestricted memory. Performance was computed as the average over 5000 runs for different random seeds (n=22) for each condition. Distribution of average performance was similar between agents using structured and unstructured representations in each of the four gridworld environments. There was no significant difference between the mean performance across random seeds for structured and unstructured representations.

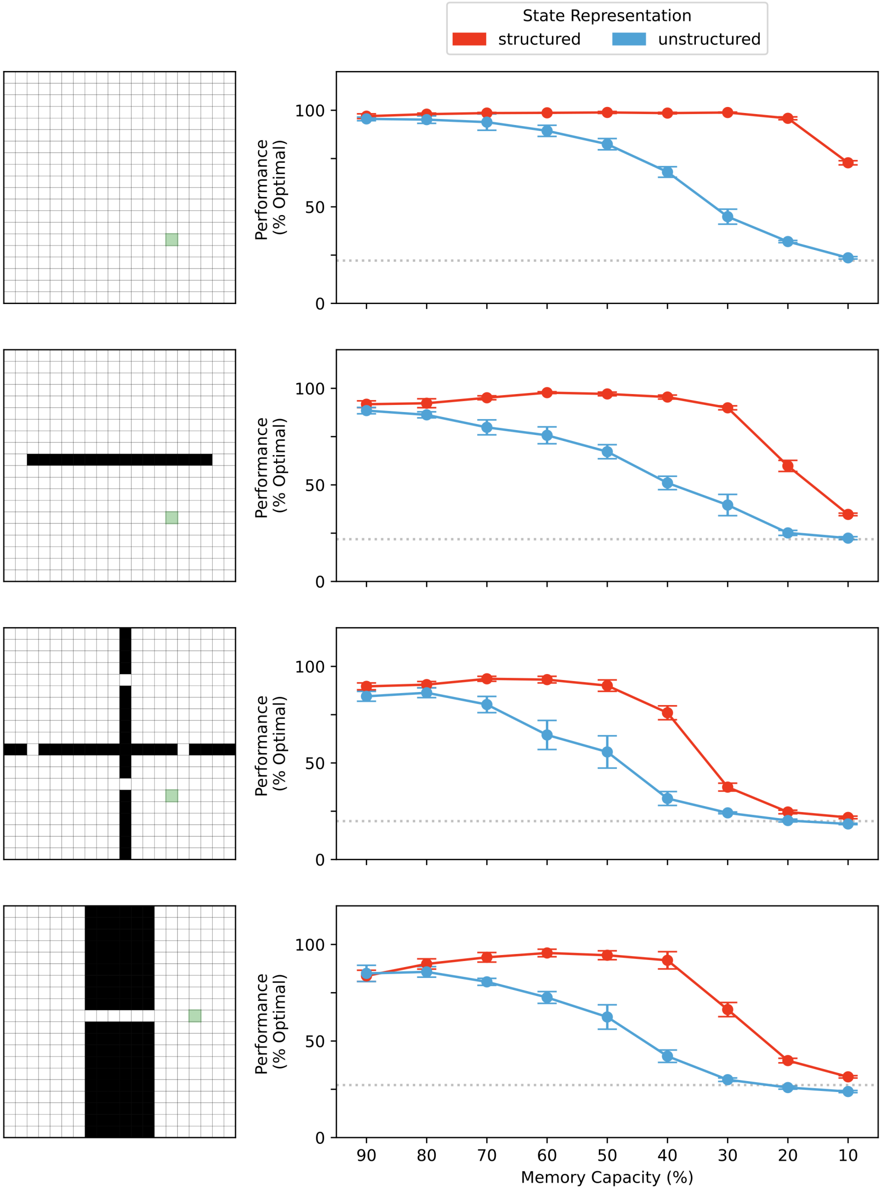

Figure S2. Average performance at memory capacity restrictions in 10% increments for agents using either structured or unstructured representations of state (n=6 for each condition). Data collected and analyzed as in Fig. 4.

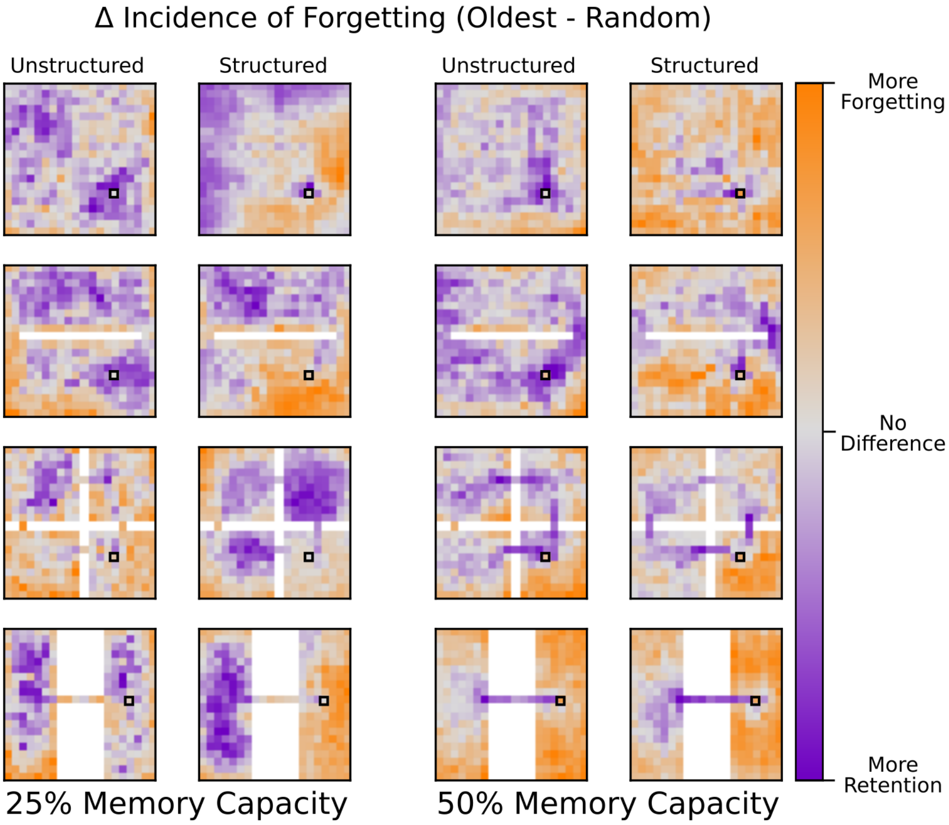

Figure S3. Results as in Fig. 8B for restriction of memory to 50% and 25% capacity. Similarly, the oldest forgetting rule showed a greater propensity for retention of bottleneck states and forgetting of peripheral states over the random forgetting rule condition, except in the case of the open field task for structured representations at 25% memory capacity. In this condition, agents using structured representations and forgetting oldest entries tended to retain memories for states more distal from the reward location at a greater rate than agents using random forgetting.
